# Supplementary material for: A highly conserved pocket on PP2A‐B56 is required for hSgo1 binding and cohesion protection during mitosis
Source: EMBO Rep. 2021 May 11;22(7):e52295. doi: 10.15252/embr.202052295 (PMC8256288; doi:10.15252/embr.202052295)
Supplement: Supplementary file 1 — Expanded View Figures PDF [file EMBR-22-e52295-s007.pdf]

Expanded View Figures

Figure EV1. Supporting information related to Fig 2.

A YFP IP from cells stably expressing YFP-hSgo2 and transfected with FLAG-B56α constructs. Representative blots are shown. FLAG-B56α signals were normalized to hSgo2 and plotted. Error bars represent SD (n = 4).

B Validation of the B56 RNAi and rescue system. Endogenous B56α was efficiently depleted 48 h after the RNAi treatment. The RNAi-resistant YFP-B56α rescue constructs were expressed approximately at the endogenous level.

C, D Localization of hSgo1(C) and hSgo2 (D) in cells depleted of B56 and expressing the indicated B56α variants. Representative images from 3 independent experiments are shown. Scale bar, 5 μm.

E Experimental protocol of the live cell imaging shown in (F).

F B56 RNAi and rescue with the indicated B56α RNAi-resistant constructs were performed. Time (min) from nuclear envelop breakdown (NEBD) is indicated. Scale bar, 15 μm.

G The time from NEBD to anaphase was measured from 2 independent live cell imaging experiments. Each circle represents an individual cell. Blue circles indicate the cells that were still arrested at the end of filming, and red circles indicate the cells that died during mitosis. The median is indicated with the red horizontal bars. A Mann–Whitney test was used for statistical comparison.

Source data are available online for this figure.

© 2021 The Authors

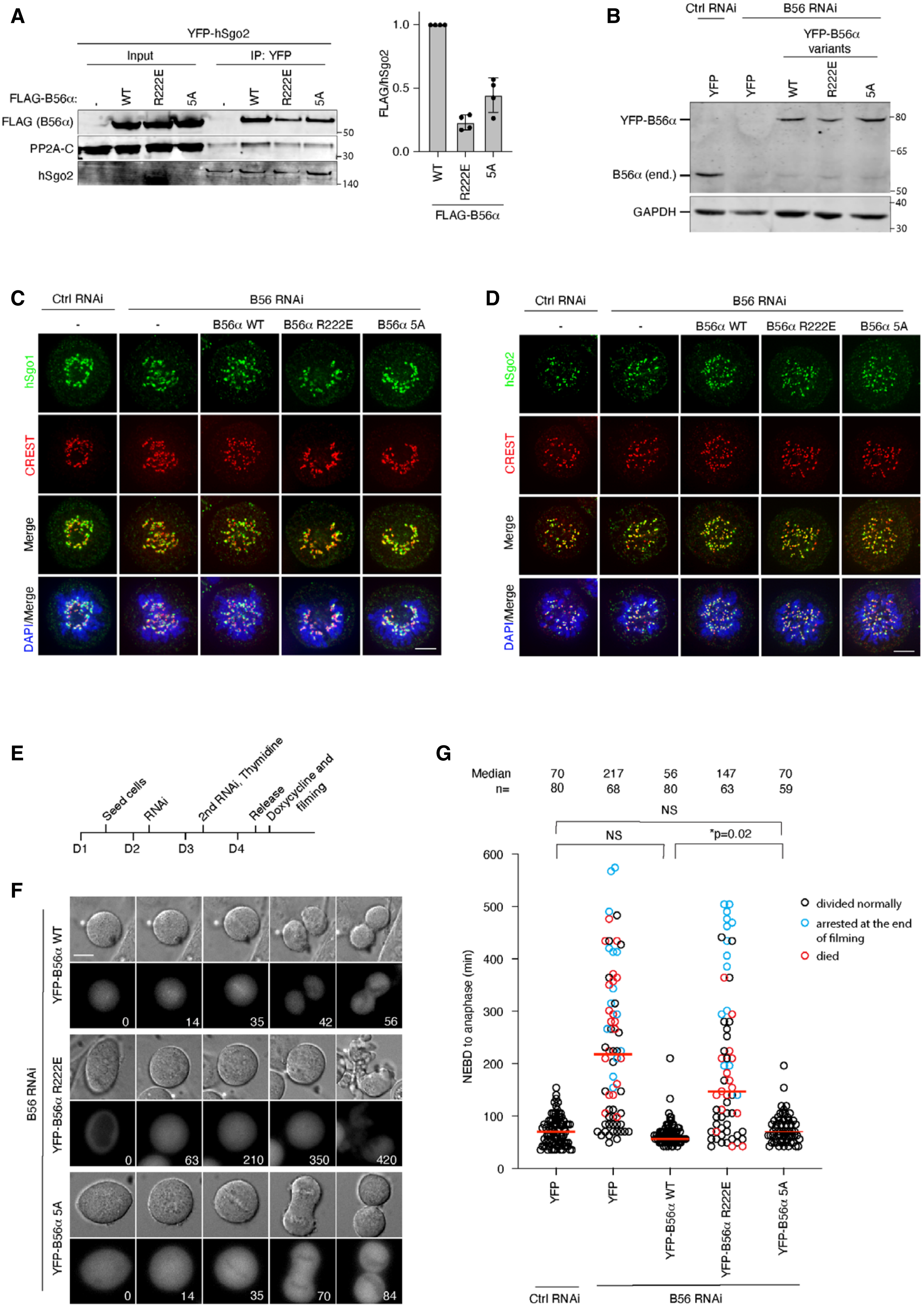
A
YFP-hSgo2
Input
IP: YFP
FLAG-B56α:
- WT R222E 5A - WT R222E 5A
FLAG (B56α)
PP2A-C
hSgo2
50
30
140
FLAG/hSgo2
1.0
0.5
0.0
WT R222E 5A
FLAG-B56α
B
Ctrl RNAi
B56 RNAi
YFP-B56α variants
YFP YFP WT R222E 5A
YFP-B56α
B56α (end.)
GAPDH
80
65
50
40
30
C
Ctrl RNAi
B56 RNAi
- - B56α WT B56α R222E B56α 5A
hSgo1
CREST
Merge
DAPI/Merge
D
Ctrl RNAi
B56 RNAi
- - B56α WT B56α R222E B56α 5A
hSgo2
CREST
Merge
DAPI/Merge
E
Seed cells
RNAi
2nd RNAi, Thymidine
Release
Doxycycline and filming
D1 D2 D3 D4
F
B56 RNAi
YFP-B56α WT
0 14 35 42 56
YFP-B56α R222E
0 63 210 350 420
YFP-B56α 5A
0 14 35 70 84
G
Median 70 217 56 147 70
n= 80 68 80 63 59
NS
NS
*p=0.02
NEBD to anaphase (min)
600
500
400
300
200
100
0
divided normally
arrested at the end of filming
died
YFP
YFP
YFP-B56α WT
YFP-B56α R222E
YFP-B56α 5A
Ctrl RNAi
B56 RNAi

Figure EV1.

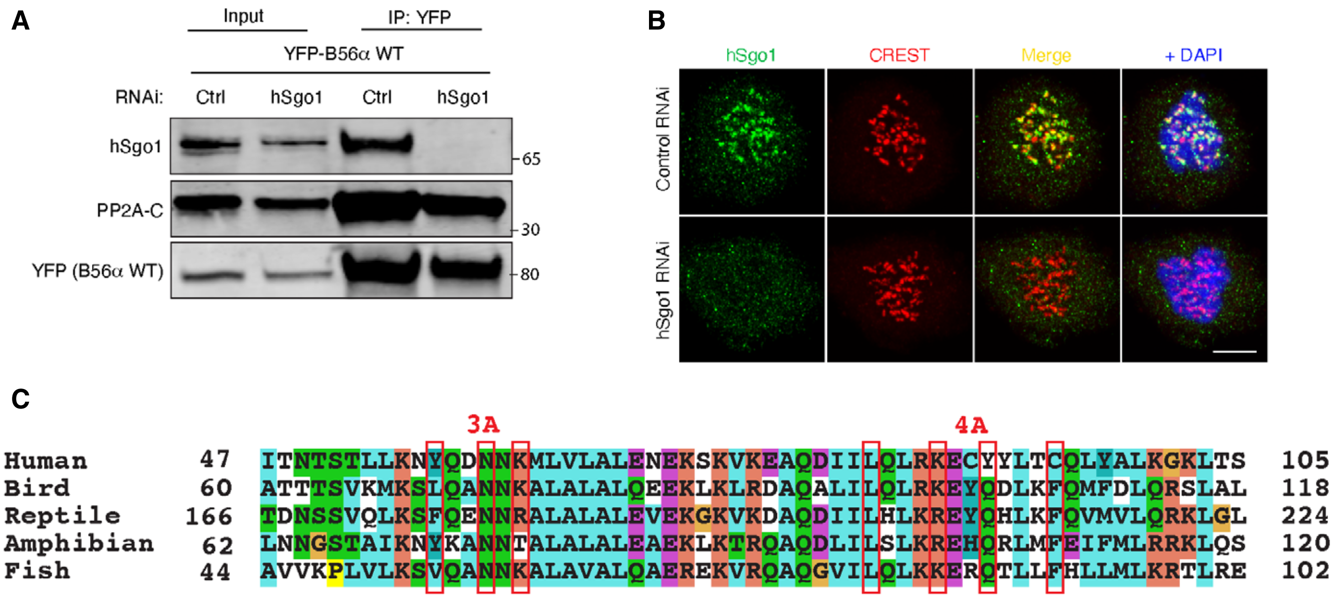

Figure EV2. Validation of hSgo1 KD efficiency and the conservation of the hSgo1 coiled-coil region.

A Validation of the hSgo1 antibody and the hSgo1 RNAi by immunoblotting. While the hSgo1 antibody detects unspecific bands in the whole cell lysates (see input), it is specific for hSgo1 after B56 IP, as the treatment with hSgo1 RNAi completely abolishes hSgo1 signal after 48h.

B Validation of the hSgo1 antibody and the hSgo1 RNAi by immunofluorescence. Representative immunofluorescent images are shown. Scale bar, 5 μm.

C The conservation of the hSgo1 coiled-coil region. The residues mutated in Sgo1 3A and 4A are indicated.

Figure EV3. Supporting information related to Fig 3F and G.

A–C Mitotic U2OS LacO cells expressing hSgo1-LacI-GFP variants or LacI-GFP (control) were stained for CPC components, Aurora B (A) and Borealin (not shown). AuroraB (B) and Borealin (C) signal intensity was quantified, normalized to GFP, and plotted. Each circle represents an individual cell, and the mean is indicated. Representative of at least 3 experiments.

D–H Mitotic U2OS LacO cells expressing hSgo1$^{1-130}$-LacI-GFP variants or LacI-GFP (control) were stained for PP2A-C (D) or CPC components, Aurora B (E) and Borealin (not shown). PP2A-C (F), AuroraB (G), and Borealin (H) signal intensity were quantified, normalized to GFP, and plotted. Each circle represents an individual cell, and the mean is indicated. Representative of at least 3 experiments.

Data information: Scale bars (in A, D–E), 5 μm.
Source data are available online for this figure.

© 2021 The Authors

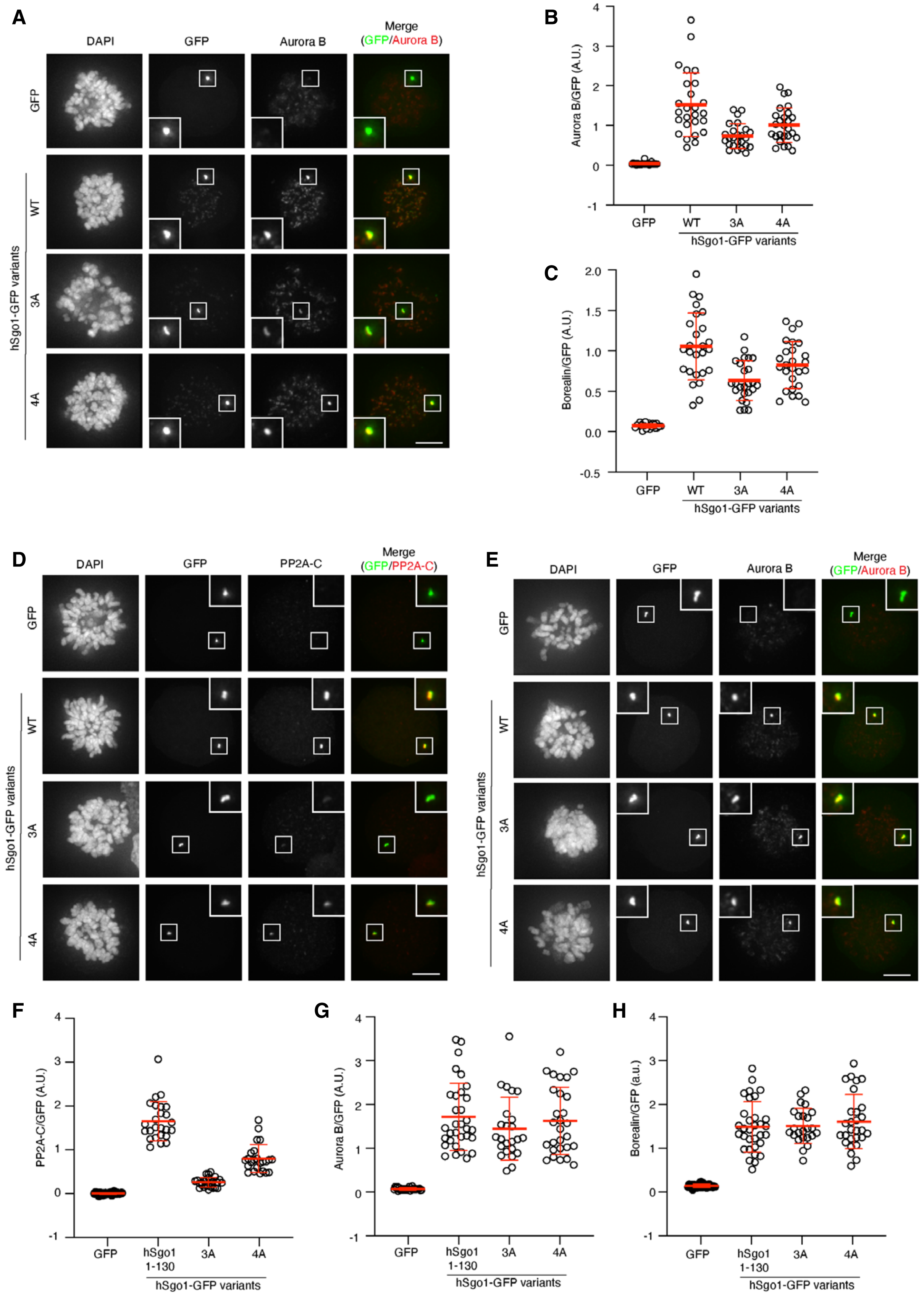
A
DAPI
GFP
Aurora B
Merge
(GFP/Aurora B)
GFP
WT
3A
4A
hSgo1-GFP variants
B
Aurora B/GFP (A.U.)
GFP
WT
3A
4A
hSgo1-GFP variants
C
Borealin/GFP (A.U.)
GFP
WT
3A
4A
hSgo1-GFP variants
D
DAPI
GFP
PP2A-C
Merge
(GFP/PP2A-C)
E
DAPI
GFP
Aurora B
Merge
(GFP/Aurora B)
F
PP2A-C/GFP (A.U.)
GFP
hSgo1 1-130
3A
4A
hSgo1-GFP variants
G
Aurora B/GFP (A.U.)
H
Borealin/GFP (a.u.)

Figure EV3.

Figure EV4. Validation of TurboID system and hSgo1KD and rescue in various conditions.

- A Blot of stable, doxycycline-inducible TurboID-hSgo1 cells treated with doxycycline and/or biotin as indicated, and probed for hSgo1 or Streptavidin.
- B Volcano plot of TurboID-hSgo1 WT cells treated or untreated with biotin. B56 regulatory subunits (2A5A-E) and centromere as well as kinetochore proteins indicated (UniProt name indicated).
- C hSgo1KD and rescue with the indicated hSgo1 RNAi-resistant constructs ± WAPL KD were performed. The time from nuclear envelop breakdown (NEBD) to anaphase was measured from the live cell imaging. Each circle represents an individual cell. Note that the hSgo1 + WAPL RNAi condition is incorporated into Fig 4G for clarity.
- D Experimental protocol of the live cell imaging with hSgo1 complementation with and without partial B56 depletion shown in (F).
- E WB showing the partial KD of all B56 isoforms.
- F hSgo1 ± partial B56 KD and rescue with the indicated hSgo1 RNAi-resistant constructs were performed. The time from nuclear envelop breakdown (NEBD) to anaphase was measured from the live cell imaging. Each circle represents an individual cell. Blue circles indicate the cells that were still arrested at the end of filming, and red circles indicate the cells that died. The median is indicated with the red horizontal bars. Representative of 2 independent experiments.

Source data are available online for this figure.

 © 2021 The Authors

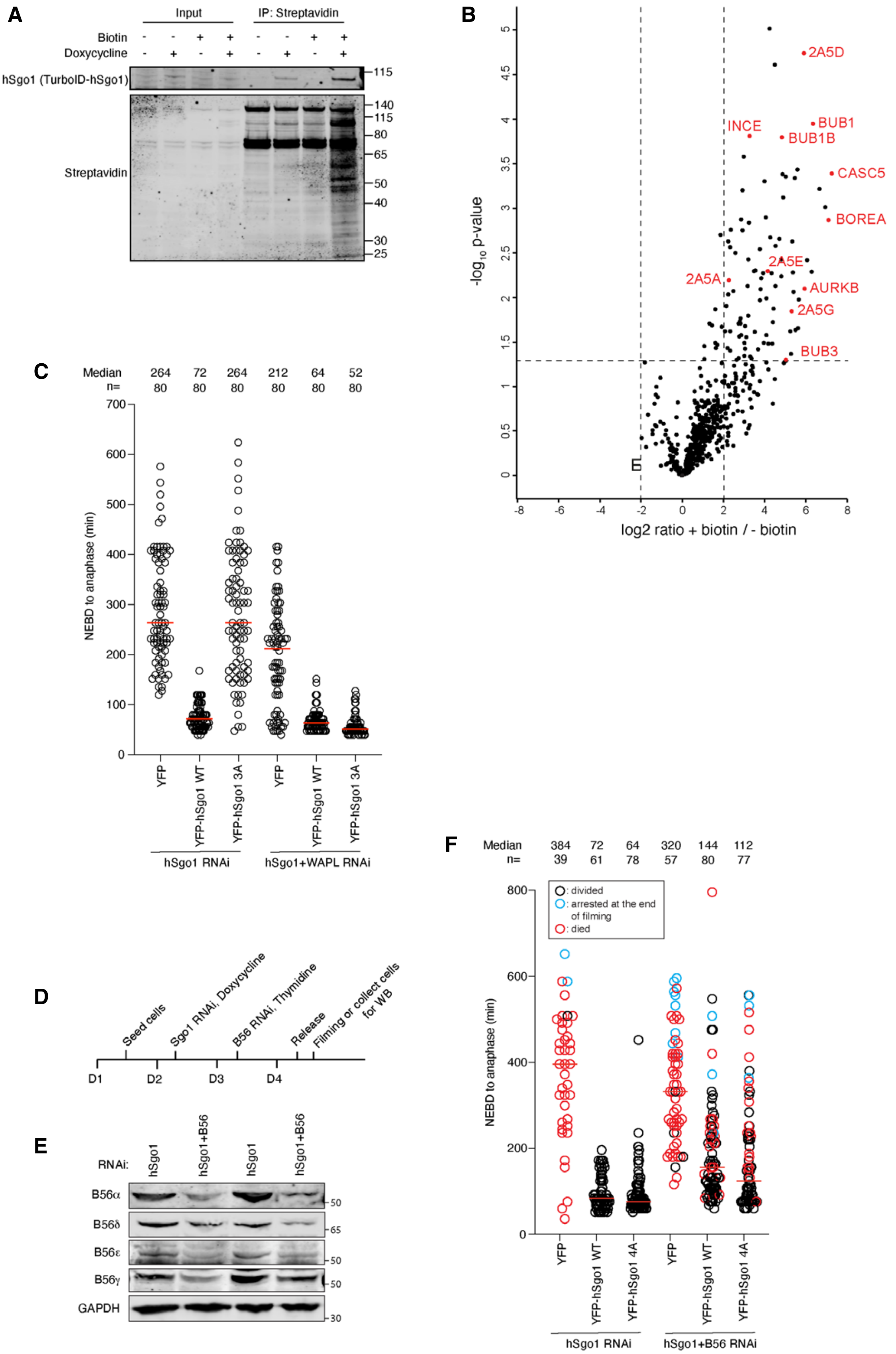
A
Input
IP: Streptavidin
Biotin
Doxycycline
hSgo1 (TurboID-hSgo1)
Streptavidin
B
-log10 p-value
log2 ratio + biotin / - biotin
2A5D
INCE
BUB1
BUB1B
CASC5
BOREA
2A5E
2A5A
AURKB
2A5G
BUB3
C
Median 264 72 264 212 64 52
n= 80 80 80 80 80 80
NEBD to anaphase (min)
YFP
YFP-hSgo1 WT
YFP-hSgo1 3A
hSgo1 RNAi
hSgo1+WAPL RNAi
D
Seed cells
Sgo1 RNAi, Doxycycline
B56 RNAi, Thymidine
Release
Filming or collect cells for WB
D1 D2 D3 D4
E
RNAi:
hSgo1
hSgo1+B56
B56α
B56δ
B56ε
B56γ
GAPDH
F
Median 384 72 64 320 144 112
n= 39 61 78 57 80 77
: divided
: arrested at the end of filming
: died
NEBD to anaphase (min)
YFP
YFP-hSgo1 WT
YFP-hSgo1 4A
hSgo1 RNAi
hSgo1+B56 RNAi

Figure EV4.
