# Supplementary material for: A highly conserved pocket on PP2A‐B56 is required for hSgo1 binding and cohesion protection during mitosis
Source: EMBO Rep. 2021 May 11;22(7):e52295. doi: 10.15252/embr.202052295 (PMC8256288; doi:10.15252/embr.202052295)
Supplement: Supplementary file 5 — Movie EV2 [file EMBR-22-e52295-s010.zip › EMBOR-2020-52295V3-Movie_EV2/Movie EV2.docx]

**Movie EV2**

Movies from time-lapse imaging of cells depleted of all B56 isoforms and expressing YFP-B56α R222E, progressing through mitosis.
